# Supplementary material for: Dietary Supplementation with Mono-Lactate Glyceride Enhances Intestinal Function of Weaned Piglets
Source: Animals (Basel). 2023 Apr 11;13(8):1303. doi: 10.3390/ani13081303 (PMC10135088; doi:10.3390/ani13081303)
Supplement: Supplementary file 1 [file animals-13-01303-s001.zip › animals-2289881-supplementary.pdf]

Table S1. The effects of dietary supplementation with mono-lactate glyceride (LG) on biochemical indices in the plasma of piglets.

Items	on day 7			on day 14			on day 21		
	Control	LG	*P*-value	Control	LG	*P*-value	Control	LG	*P*-value
ALT (U/L)	36.20 ± 5.58	36.21 ± 10.30	0.998	43.27 ± 6.65	37.03 ± 8.52	0.084	44.53 ± 2.53[b]	54.15 ± 12.40[a]	0.027
AST (U/L)	37.90 ± 7.20	46.72 ± 12.87	0.075	40.05 ± 9.89	37.07 ± 7.70	0.462	64.42 ± 18.95	63.24 ± 10.78	0.866
TBIL (μmol/L)	2.64 ± 0.48	2.70 ± 0.50	0.787	2.51 ± 0.26[a]	2.22 ± 0.11[b]	0.005	4.21 ± 1.32	4.82 ± 1.11	0.278
TP (g/L)	49.68 ± 2.70[a]	46.95 ± 2.11[b]	0.022	49.97 ± 2.91	49.11 ± 0.45	0.368	50.06 ± 4.18	51.39 ± 2.80	0.414
ALB (g/L)	28.43 ± 1.74	27.15 ± 1.43	0.089	28.16 ± 1.91[a]	26.60 ± 0.99[b]	0.035	28.70 ± 2.17	28.65 ± 1.53	0.953
CHOL (mmol/L)	1.77 ± 0.29[a]	1.52 ± 0.14[b]	0.025	1.96 ± 0.16	1.96 ± 0.19	0.939	1.84 ± 0.27	1.92 ± 0.23	0.509
BUN (mmol/L)	1.76 ± 0.34[a]	2.39 ± 0.54[b]	0.006	2.29 ± 0.66	1.91 ± 0.32	0.114	2.21 ± 0.59	1.93 ± 0.29	0.192
ALP (U/L)	223.47 ± 71.47[b]	304.80 ± 68.74[a]	0.018	236.23 ± 53.35	234.92 ± 53.29	0.957	234.63 ± 55.81	248.05 ± 65.99	0.629
CK (U/L)	485.11 ± 156.95	539.22 ± 174.40	0.475	962.31 ± 304.59[a]	491.48 ± 175.78[b]	<0.001	1588.20 ± 399.98[a]	1249.30 ± 257.95[b]	0.037
GGT (U/L)	36.23 ± 8.94[a]	27.33 ± 5.57[b]	0.016	30.91 ± 3.04[a]	25.97 ± 3.04[b]	0.002	28.42 ± 5.44	25.45 ± 3.03	0.149

Values are mean and SD, n = 8. [a, b] Values within a row with different letters differ ($P < 0.05$). ALP, alkaline phosphatase; BUN, blood urea nitrogen; ALT, alanine aminotransferase; TP, total protein; CHOL, total cholesterol; GGT, glutamyl transpeptidase; TBIL, total bilirubin; ALB, albumin; CK, creatine kinase.

Table S2. The effects of mono-lactate glyceride (LG) supplementation on the mRNA levels of genes related to intestinal mucositis in piglets.

Items	Jejunum			Ileum			Colon		
	Control	LG	*P*-value	Control	LG	*P*-value	Control	LG	*P*-value
IL-1β	1.00 ± 0.24[b]	1.58 ± 0.26[a]	<0.001	1.00 ± 0.21[b]	2.29 ± 0.60[a]	<0.001	1.00 ± 0.26	0.81 ± 0.18	0.118
IL-4	1.00 ± 0.26	0.89 ± 0.11	0.270	1.00 ± 0.27[b]	1.83 ± 0.38[a]	<0.001	1.00 ± 0.17	0.86 ± 0.21	0.152
IFN-γ	1.00 ± 0.22[b]	1.29 ± 0.26[a]	0.031	1.00 ± 0.17[b]	1.26 ± 0.25[a]	0.027	1.00 ± 0.22[a]	0.72 ± 0.17[b]	0.014
CCL-2	1.00 ± 0.15	1.00 ± 0.27	0.991	1.00 ± 0.21[b]	1.47 ± 0.36[a]	0.006	1.00 ± 0.25	0.81 ± 0.20	0.113
CXCL-9	1.00 ± 0.23	1.23 ± 0.25	0.076	1.00 ± 0.25[a]	0.77 ± 0.09[b]	0.032	1.00 ± 0.20	1.11 ± 0.26	0.354

Values are mean and SD, n = 8. [a,b] Values within a row with different letters differ ($P < 0.05$).
