# Supplementary figures and images for: Dietary Supplementation with Mono-Lactate Glyceride Enhances Intestinal Function of Weaned Piglets
Source: Animals (Basel). 2023 Apr 11;13(8):1303. doi: 10.3390/ani13081303 (PMC10135088; doi:10.3390/ani13081303)

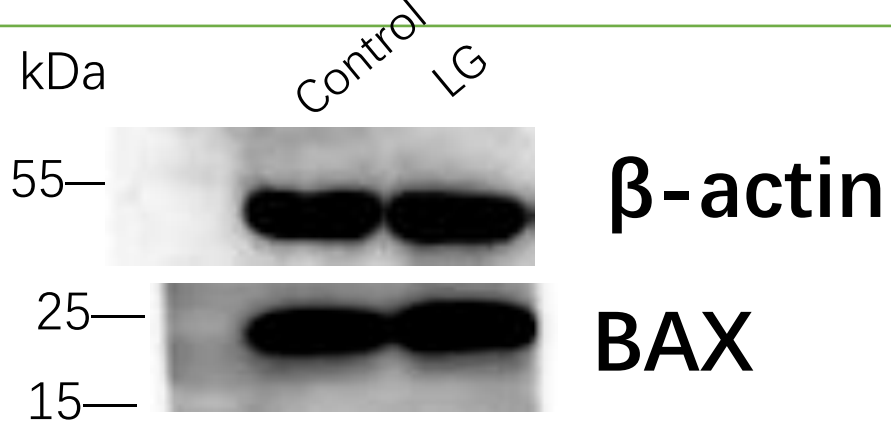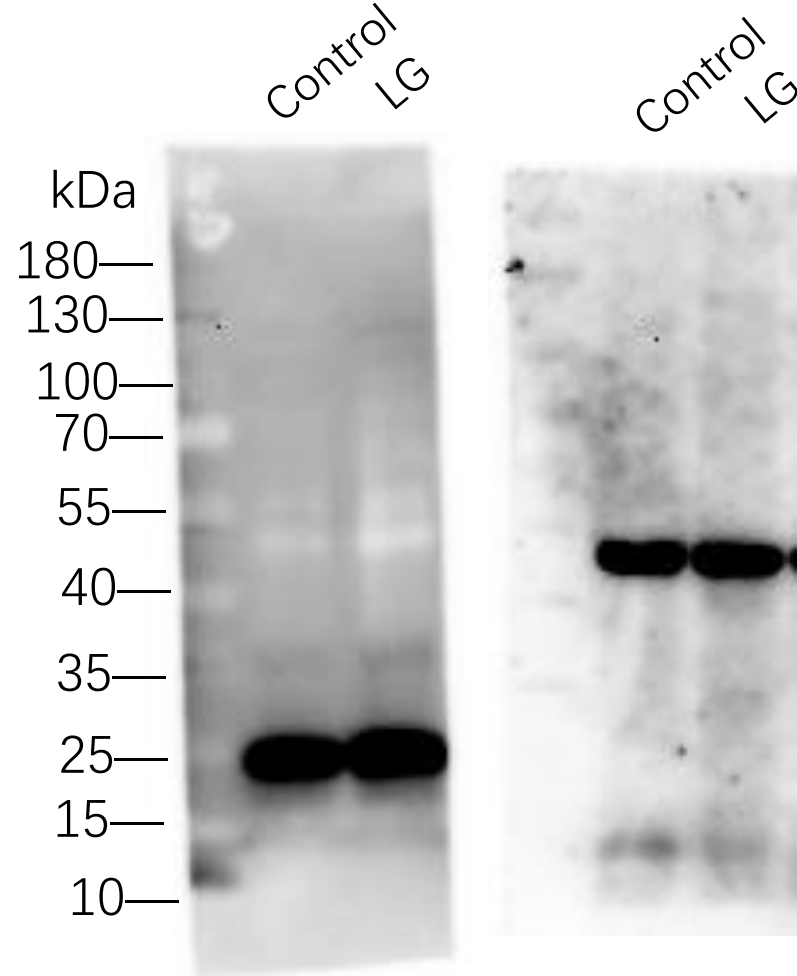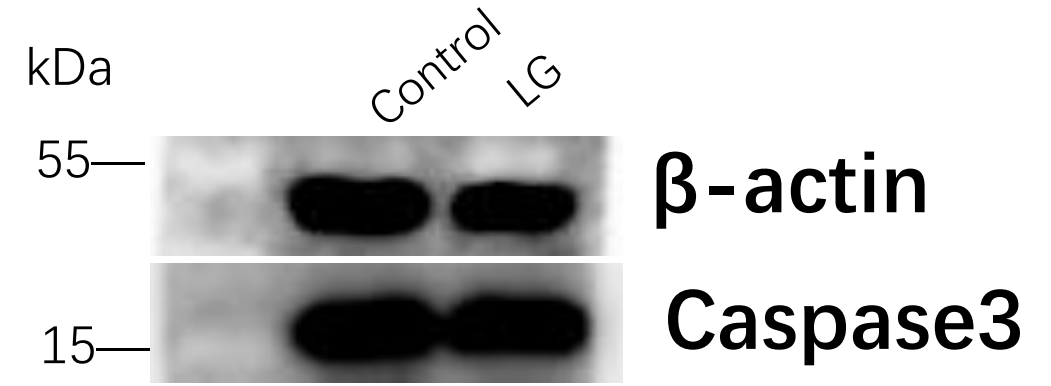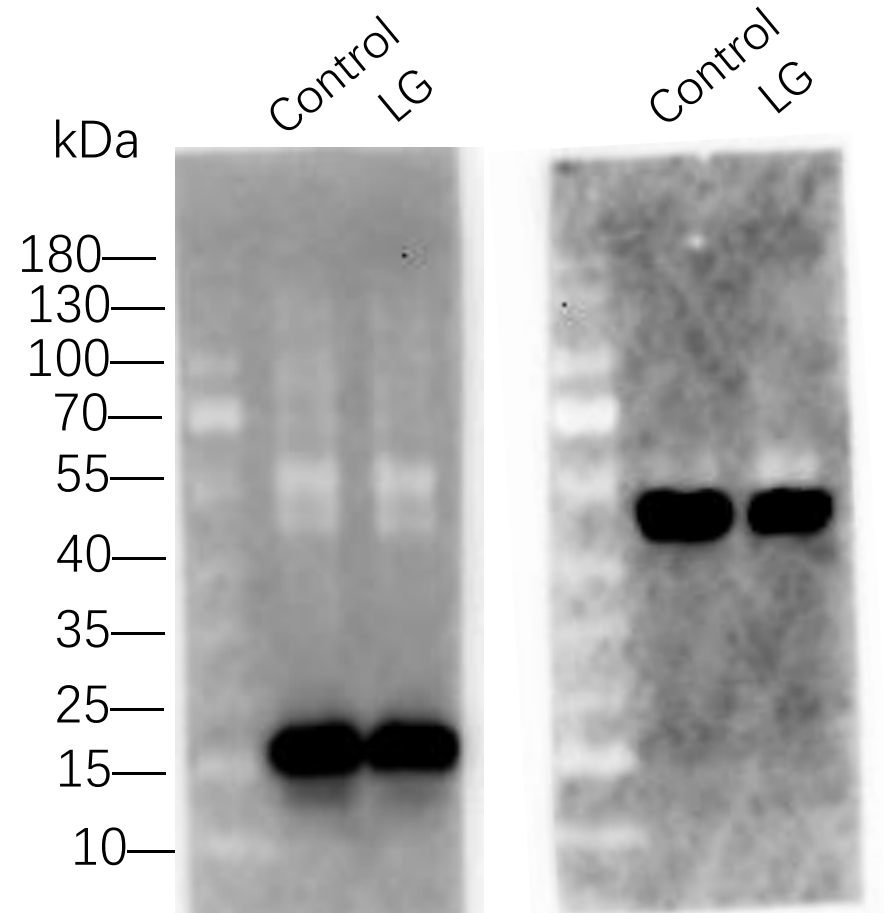

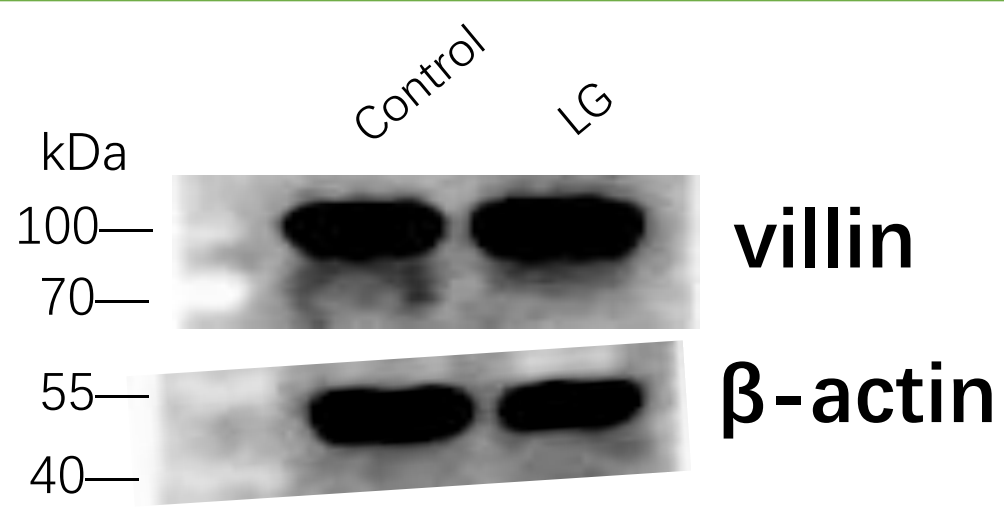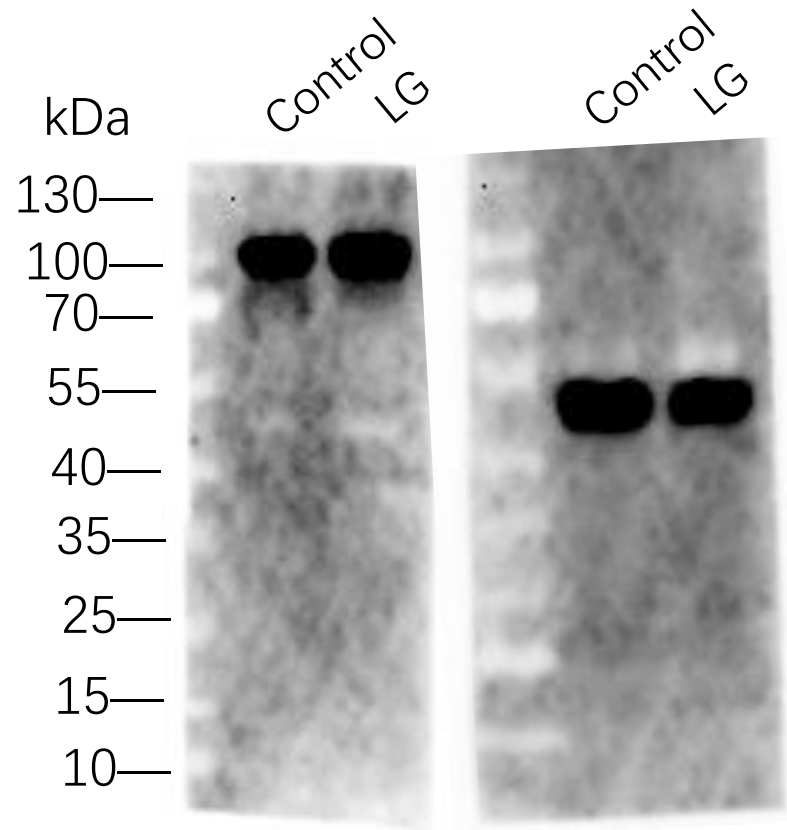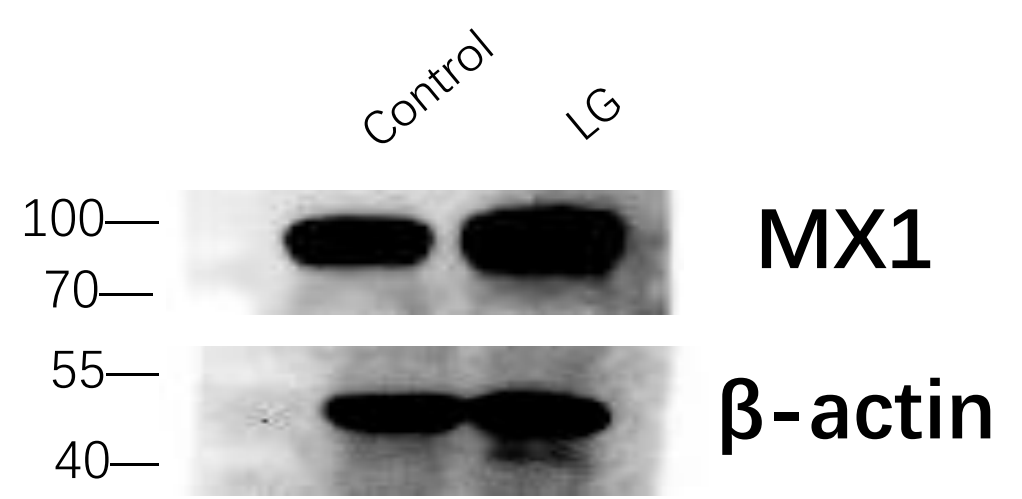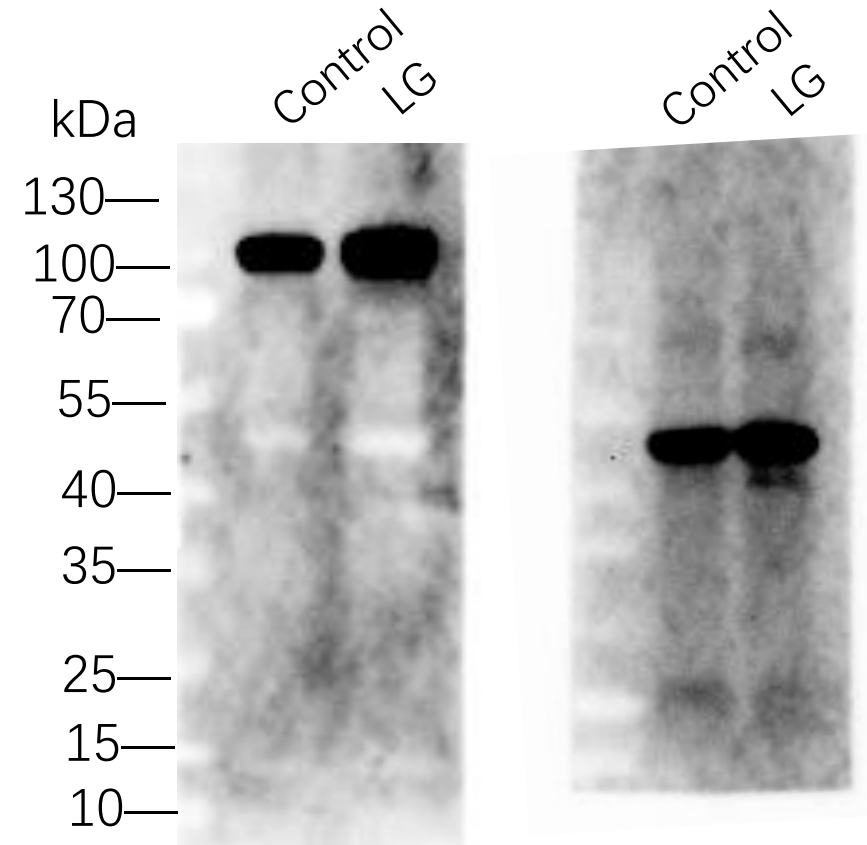

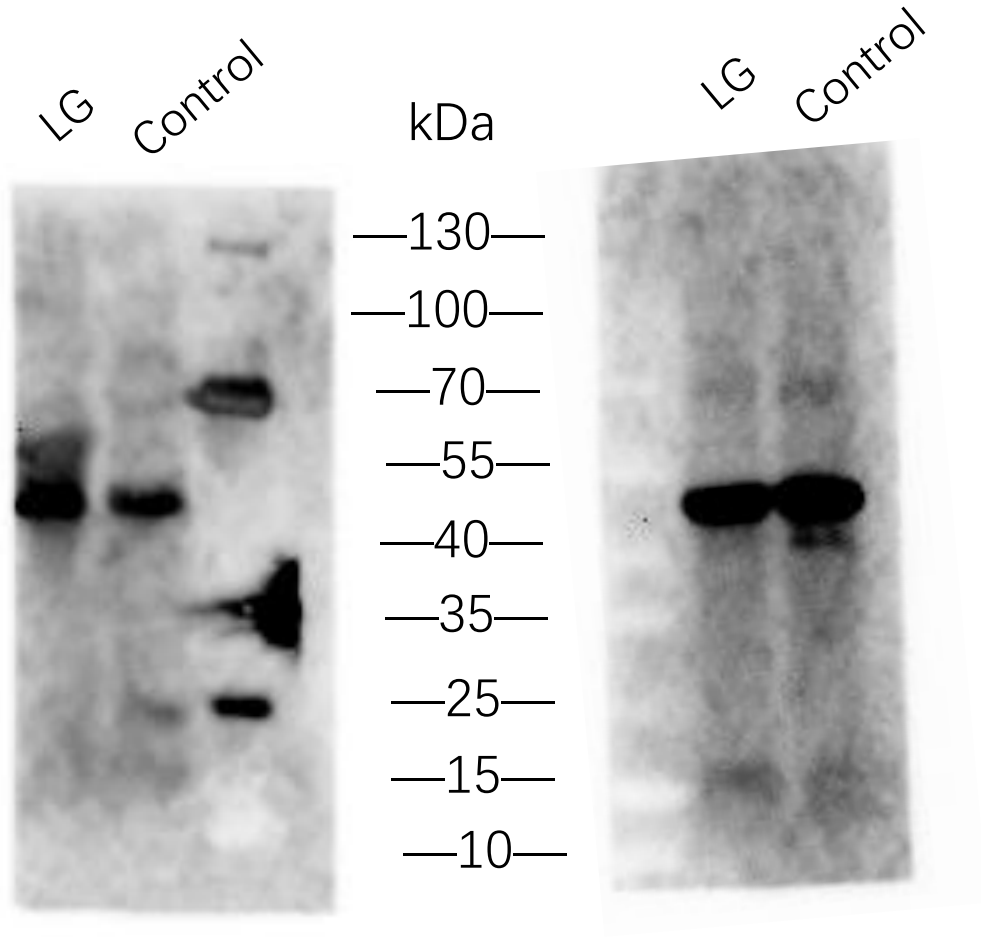

**Occludin**

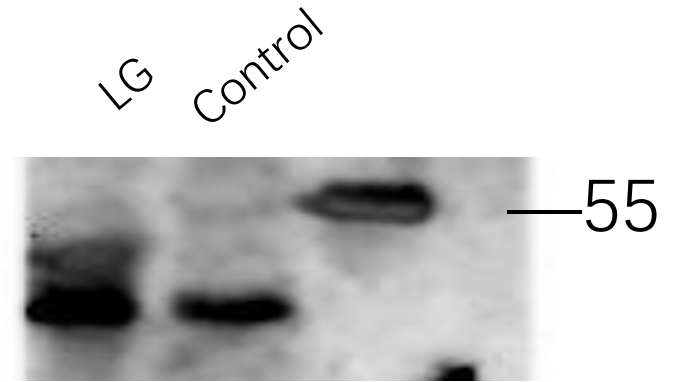

55—  
40—

**β-actin**

Supplement: Supplementary file 1 [file animals-13-01303-s001.zip › animals-2289881-supplementary-Figure S1.pdf]
